# *Staphylococcus lugdunensis* Endophthalmitis: Case Series and Literature Review

**DOI:** 10.3390/antibiotics11111485

**Published:** 2022-10-26

**Authors:** Kuan-Jen Chen, Ming-Hui Sun, Andrew S. H. Tsai, Chi-Chin Sun, Wei-Chi Wu, Chi-Chun Lai

**Affiliations:** 1 Department of Ophthalmology, Chang Gung Memorial Hospital, Linkou branch, Taoyuan 333, Taiwan; 2 College of Medicine, Chang Gung University, Taoyuan 333, Taiwan; 3 Singapore National Eye Centre, Singapore 168751, Singapore; 4 Department of Ophthalmology, Chang Gung Memorial Hospital, Keelung 204, Taiwan

**Keywords:** antibiotic susceptibility, endophthalmitis, *Staphylococcus lugdunensis*, vancomycin, vitrectomy

## Abstract

*Staphylococcus lugdunensis* endophthalmitis is an uncommon intraocular infection with potentially visually devastating consequences. *S. lugdunensis* endophthalmitis have been reported following cataract surgery, trauma, intravitreal injections of anti-vascular endothelial growth factor agents and dexamethasone implant. We report four cases of postoperative *S. lugdunensis* endophthalmitis after cataract extraction (three patients) and combined pars plana vitrectomy and cataract extraction (one patient). The onset of presentation of endophthalmitis was acute (within 2 weeks) in two patients, subacute (2 to 6 weeks) in one patient, and chronic (more than 6 weeks) in one patient. All patients had presenting visual acuity (VA) of hand motions or worse and were treated with pars plana vitrectomy with intravitreal antibiotics. The final VA was 20/50 in two patients, 4/200 in one patient with pre-existing myopic maculopathy, and no light perception in one patient with retinal detachment. In antibiotic susceptibility testing, *S. lugdunensis* isolates were resistant to penicillin (3/4, 75%), but all were susceptible to vancomycin, oxacillin, teicoplanin, tigecycline, and sulfamethoxazole-trimethoprim. *S. lugdunensis* may be associated with acute or chronic endophthalmitis. Favorable visual outcomes can be achieved with prompt diagnosis and management.

## 1. Introduction

Bacterial endophthalmitis is the most feared complication of intraocular surgery that can result in severe visual loss. Acute postoperative endophthalmitis is mainly caused by patients’ own flora on the ocular surface, with the majority being associated with *Staphylococcus* species. The incidence of postoperative endophthalmitis ranged from 0.02 to 0.2% after cataract extraction and from 0.016 to 0.083% after intravitreal injections of anti-vascular endothelial growth factor (VEGF) agents or corticosteroids [1,2]. Management of postoperative bacterial endophthalmitis is important to save the vision and decrease the burden of this complication. In the Endophthalmitis Vitrectomy Study (EVS), conventional culture techniques revealed that coagulase-negative staphylococci (CNS) accounted for 70% of all cases, and 9 (3.6%) of 250 intraocular specimens grew *Staphylococcus lugdunensis* [3,4]. The EVS also reported that *S. lugdunensis* accounted for a total of 5.9% of coagulase-negative Staphylococcus specimens cultured from both intraocular and extraocular sites, whereas S. epidermidis accounted for 81.9% [4]. *S. lugdunensis* is a Gram-positive, coagulase-negative bacterium, and can cause infective endocarditis, bone and joint infections, and skin and soft tissue infections [5,6,7]. Several reports of patients with *S. lugdunensis* endophthalmitis have been reported following cataract surgery, trauma, and intravitreal injections of anti-VEGF agents and dexamethasone implant [4,8,9,10,11,12,13], but it still remains an infrequent cause of post-operative endophthalmitis. In this study, we investigate the clinical presentation, antibiotic susceptibilities, and treatment outcomes of all patients with endophthalmitis caused by *S. lugdunensis* at a tertiary referral center in Northern Taiwan. We also conducted a literature review and compared the clinical characteristics with our study.

## 2. Methods and Materials

This study is a retrospective, consecutive case series of patients diagnosed with *S. lugdunensis* endophthalmitis from January 2009 to April 2019 at a tertiary referral center in Northern Taiwan. The institutional review board of Chang Gung Memorial Hospital in Taoyuan, Taiwan, approved this retrospective study protocol (201900614B0C601, 10 August 2019) and waived the need for written informed consent by these patients. All clinical procedures were conducted according to the principles of the Declaration of Helsinki. A review of all patients with a diagnosis of acute-onset endophthalmitis at the Chang Gung Memorial Hospital Microbiology Department was performed. Patients were included in this study if vitreous and/or aqueous samples taken at the time of diagnosis grew isolates of *S. lugdunensis*. The availability of medical records used electronic systems. Data collected and reviewed included demographic information, medical history, presenting signs and symptoms, duration of symptoms before diagnosis of endophthalmitis, intervals between event, and diagnosis of endophthalmitis, culture sites, antibiotic sensitivities and resistance patterns, management administered, and final visual acuity (VA). The doses of intravitreal agents were as follows: vancomycin, 1 mg/0.1 mL; ceftazidime, 2.25 mg/0.1 mL; and dexamethasone, 0.4 mg/0.1 mL. Poor visual outcomes were defined as VA worse than 20/200.

All microbiological investigations were performed at the Microbiology Department of Chang Gung Memorial Hospital, Taoyuan, Taiwan. Bacterial culture isolates were identified using conventional microbiological methods and matrix-assisted laser desorption/ionization-time of flight mass spectrometry (MALDI-TOF-MS). Conventional microbiological methods included Gram staining and biochemical tests. In MALDI-TOF-MS, automated measurement of the spectrum and comparative analysis against reference bacteria spectra were performed using an Ultraflextreme mass spectrometer and in MALDI Biotyper 3.0 software (Bruker Daltonics, Karlsruhe, Germany). The reliability of identification in the MALDI Biotyper system was expressed in terms of points. A log(score) of ≥2.0 indicated identification to the species level. The antibiotic susceptibility testing results were based on the choice of antibiotics tested in our hospital during the study period. The isolates were tested for susceptibility to various antibiotics using the Kirby–Bauer disc diffusion method on Mueller–Hinton blood agar. Clinical and Laboratory Standards Institute (Wayne, PA, USA) standards were used for interpretation and quality control for each corresponding year.

## 3. Results

**Figure 1:**
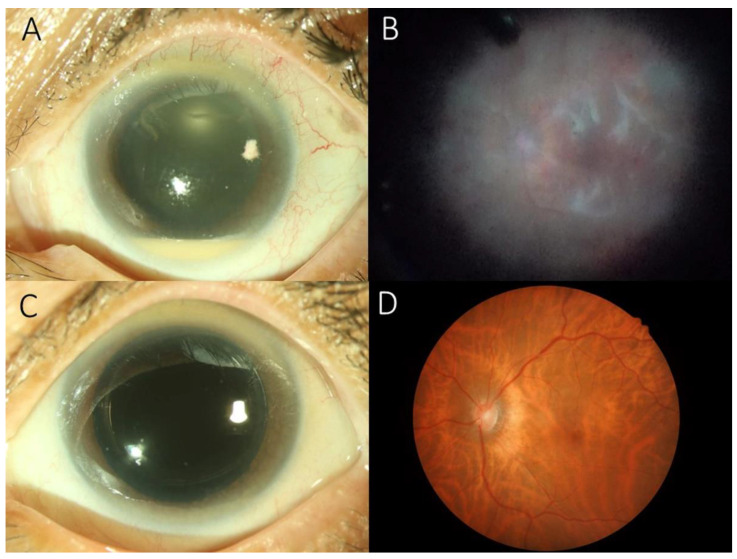
(**A**) Slit-lamp biomicroscopic examination showing corneal edema with a 2-mm hypopyon in patient 4. (**B**) During pars plana vitrectomy, posterior pole showing diffuse retinal vasculitis. (**C**) Four weeks later, Slit-lamp biomicroscopic examination showing clear cornea without hypopyon. (**D**) Fundoscopic examination revealing myopic tessellated retina with resolution of retinal vasculitis.

A total of four patients were diagnosed with *S. lugdunensis* endophthalmitis during the study period. The mean age of patients was 71.0 ± 3.5 years (range: 67 to 75 years). All patients were female. Table 1 shows the clinical summary of the patients. Based on the interval between event (time of surgery) and diagnosis of endophthalmitis, the onset of presentation of endophthalmitis was classified as acute (with 2 weeks) in two patients, subacute (2 to 6 weeks) in one patient, and chronic (more than 6 weeks) in one patient. Ocular B-mode ultrasonography revealed moderate to dense, hyper-reflective echogenicity in vitreous cavity of all four patients. Fundal examination during vitrectomy showed diffuse retinal vasculitis and hemorrhages (Figure 1) in all patients. One patient (patient 3) had dense posterior pole preretinal exudates and retinal detachment. Three patients were successfully managed with pars plana vitrectomy. One patient developed retinal detachment even after vitrectomy. All patients were treated with topical either 0.5% levofloxacin or 2.5% vancomycin and 1 % prednisolone acetate after vitrectomy, but they did not receive oral antibiotics. In last visit, two eyes achieved favorable visual outcomes with 20/50 vision, but one eye had a final visual acuity of 4/200 that was limited by pre-existing myopic maculopathy.

### Antibiotic Susceptibility Testing

Table 2 presents the antibiotic susceptibility of *S. lugdunensis* isolates. Most *S. lugdunensis* isolates were resistant to penicillin (3/4), but all were susceptible to vancomycin, which is currently being used empirically to treat Gram-positive bacterial endophthalmitis. The isolates were also susceptible to oxacillin, teicoplanin, tigecycline, and sulfamethoxazole-trimethoprim. One isolate was resistant to clindamycin and erythromycin.

## 4. Discussion

*S. lugdunensis* is a constituent of the human normal skin flora and an infrequent human pathogen. Early studies established *S. lugdunensis* as a skin, nasal cavity, conjunctival commensal [6,14,15,16]. It was often misidentified as *S. aureus*, but this has been rectified by recent routine use of matrix-assisted laser desorption ionization-time of flight mass spectrometry (MALDI-TOF MS) in diagnostic laboratories [16]. *S. lugdunensis* represents an ambiguous staphylococcal species, and, since its first description in 1988 [6], there is growing evidence that it should be considered a dangerous opportunistic pathogen rather than just as a harmless skin commensal [6,7]. The aggressive nature of *S. lugdunensis* may be related to its tendency to form a biofilm, which has a role in bacterial colonization and interferes with the phagocytosis-associated activities of neutrophils and the production of enzymes (esterase, fatty acid-modifying enzymes, protease, and lipase) [6]. Without doubt, *S. lugdunensis* represents the most aggressive species of CNS [16]. Compared with patients with other CNS endophthalmitis, patients with *S. lugdunensis* endophthalmitis often had a worse final visual outcome and a higher frequency of post-vitrectomy retinal detachment [8].

Previous studies showed that cases of *S. lugdunensis* endophthalmitis occurred within 3 weeks. However, in our series, two patients occurred within 2 weeks of surgery, and another two patients developed endophthalmitis on postoperative day 41 and 83. This could represent delayed onset endophthalmitis or secondary infection from unknown reasons. During vitrectomy, diffuse retinal vasculitis and hemorrhages were identified in all patients. One patient (patient 3) had condensed posterior pole preretinal exudates and retinal detachment, and became phthisic in last visit. We previously reported that acute endophthalmitis with severe vitritis are typically associated with condensed posterior pole preretinal exudates, which might be associated with poor visual outcomes [17].

The comparisons of our study with the literature are listed in Table 3. In a study of five patients with acute postoperative *S. lugdunensis* endophthalmitis following cataract surgery, Chiquet et al. [8] reported two patients with good visual outcome. One patient had intravitreal antibiotics alone and the other received both intravitreal antibiotics and vitrectomy. Three patients had poor visual outcomes after pars plana vitrectomy which resulted in retinal detachment [8]. In another report of six patients with acute-onset *S. lugdunensis* endophthalmitis caused by surgery (cataract surgery in three patients and intravitreal injection in one) or trauma (two patients), Garoon et al. [13] reported that the final visual acuity was ≥20/400 in 6/6 (100%) of eyes and ≥20/40 in 3/6 (50%) of cases after receiving intravitreal antibiotics (three patients) or pars plana vitrectomy (three patients). In previous reports [8,9,10,12] regarding *S. lugdunensis* endophthalmitis after intravitreal injection of anti-VEGF or dexamethasone implant (Ozurdex), a favorable visual outcome (≥20/200) was achieved in four patients receiving intravitreal antibiotics or pars plana vitrectomy. In our study, three eyes were successfully managed with pars plana vitrectomy, and two eyes achieved favorable visual outcomes. Given the mixed results, it is possible that pars plana vitrectomy may have impacted the final visual outcome in patients with worse presenting VA, and intravitreal injections of antibiotics alone could have resulted in favorable outcomes in patients with better presenting VA.

In our study, all *S. lugdunensis* isolates were susceptible to vancomycin, but most isolates (75%) were resistant to penicillin. All isolates were also susceptible to oxacillin, teicoplanin, tigecycline, and sulfamethoxazole-trimethoprim. In previous studies in patients with *S. lugdunensis* endophthalmitis, all isolates identified were susceptible to vancomycin [8,9,13]. Garoon et al. reported three of six isolates of *S. lugdunensis* demonstrated resistance to oxacillin and one isolate demonstrated resistance to ciprofloxacin [13]. All six isolates were susceptible to levofloxacin, and minimum inhibitory concentration values of four isolates were 0.25 μg/mL [13]. The susceptibility testing of fluoroquinolones for *S. lugdunensis* was not performed in our hospital; therefore, there was no susceptibility testing data available for levofloxacin.

The study has some limitations. First, this was a single-center retrospective small case series. Second, *S. lugdunensis* isolates were not routinely tested for fluoroquinolones at our hospital. Third, follow-up periods were not standardized. Despite these limitations, our findings contribute to the literature in providing detailed analysis and review of *S. lugdunensis* endophthalmitis.

In conclusion, *S. lugdunensis* may be associated with acute or chronic endophthalmitis. Prompt diagnosis and management can result in favorable visual outcomes. Even though vancomycin-resistant Staphylococcus species are emerging in certain regions, vancomycin provides consistent coverage against *S. lugdunensis,* which has no known resistance at present, and remain a first line agent in treating endophthalmitis caused by CNS [18,19].

## Figures and Tables

**Table 1:** Clinical summary of patients with postoperative endophthalmitis caused by *S. lugdunensis*.

No.	Sex/Age /Eye	Interval (Days)	Symptom (Days)	Etiology	IOP (mmHg)	Culture Sites	Systemic Diseases	Initial VA	Treatment		Final VA	Other Eye Condition	Follow-Up (Months)
									Primary	Secondary			
1	F/67/OS	7	2	CE + IOL	10	AC, V	CHF, HT, DM, CVA	HM 10 cm	PPV + IVAB	IVAB(1M)	20/50	M-NPDR	36
2	F/75/OD	41	1	PPV + CE + IOL	12	AC, V		HM 30 cm	PPV + IVAB		4/200	MM	16
3	F/74/OD	83	3	CE + IOL	30	AC, V	Goiter	LP 10 cm	PPV + IVAB	IVAB(3D)	NLP	RD	3
4	F/68/OS	3	1	CE + IOL	7	V	Miller-Fisher syndrome	HM 40 cm	PPV + IVAB		20/50		54

AC, anterior chamber; CE, cataract extraction; CHF, congestive heart failure; CVA, cerebrovascular accident; D, day; DM, diabetes mellitus; HM, hand motions; IOL, intraocular lens implantation; IOP, intraocular pressure; IVAB, intravitreal antibiotics; LP, light perception; M, month; MM, high myopic maculopathy with foveoschisis; M-NPDR, moderate non-proliferative diabetic retinopathy; NLP, no light perception; PPV, pars plana vitrectomy, RD, retinal detachment; HT, hypertension; V, vitreous.

**Table 2:** Antibiotic susceptibility testing results of *Staphylococcus lugdunensis* isolates.

Antibiotics	Patient			
	1	2	3	4
Penicillin	R	R	S	R
Oxacillin	S		S	S
Teicoplanin	S	S	S	S
Vancomycin	S	S	S	S
Tigecycline	S	S	S	S
SMX/TMP	S	S	S	S
Linezolid	S			
Clindamycin	S	S	R	S
Erythromycin	S	S	R	S

R, resistant; S, susceptible; SMX/TMP, sulfamethoxazole-trimethoprim.

**Table 3:** Comparisons of published and current studies of *Staphylococcus lugdunensis* endophthalmitis.

No.	Author	Nation	Year (Published)	No. of Eyes	Interval (Days)	Etiology	Initial VA	VAN	Treatment	Final VA	Cause of Poor VA
1	Chiquet et al. [8]	France	2004–2005 (2007)	5	7	CE	HM	S	IVAB, PPV	20/40	
					6	CE	LP	S	IVAB, PPV	HM	RD
					5	CE + IOL	LP	S	IVAB, PPV	NLP	RD
					12	CE + IOL	20/100	S	IVAB	20/20	
					7	CE + IOL	HM	S	IVAB, PPV	CF	RD
2	Garoon et al. [13]	US	1990–2017 (2018)	6	10	CE + IOL	CF	S	IVAB	20/30	
					8	CE + IOL	HM	S	IVAB	20/40	
					21	IVI	CF	S	IVAB	20/40	
					6	CE + IOL	HM	S	IVAB, PPV	20/40	
					2	Trauma	HM	S	PPV + IVAB	20/400	
					3	Trauma	LP	S	PPV + IVAB	20/100	
3	Salceanu et al. [10]	UK	2019	1	4	IVI (Ozurdex)	CF	NA	PPV + IVAB	20/120	
4	Wani et al. [12]	Kuwait	NA (2016)	1	3	IVI (Avastin)	20/150	NA	IVAB	20/30	
5	Veliev et al. [11]	Turkey	NA (2022)	1	NA	postoperative	NA	NA	NA	NA	
6	Murad-Kejbou et al. [9]	US	2011–2012 (2014)	3	2	IVI (Lucentis)	LP	S	PPV + IVAB	HM	
					3	IVI (Lucentis)	20/200	S	IVAB	20/200	
					2	IVI (Avastin)	20/200	S	IVAB, PPV	20/100	
7	Bannerman et al. [4]	US	1990–1996 (1997)	4	NA	postoperative	NA	NA	NA	NA	
8	Current study Chen et al.	Taiwan	2010–2019	4	7	CE + IOL	HM	S	PPV + IVAB	20/50	
					41	PPV + CE + IOL	HM	S	PPV + IVAB	4/200	MM
					83	CE + IOL	LP	S	PPV + IVAB	NLP	RD
					3	CE + IOL	HM	S	PPV + IVAB	20/50	

CE, cataract extraction; CF, counting fingers; HM, hand motions; IOL, intraocular lens implantation; IVAB, intravitreal antibiotics; IVI, intravitreal injection; LP, light perception; MM, myopic maculopathy; NA, not available; NLP, no light perception; PPV, pars plana vitrectomy, RD, retinal detachment; S, susceptible, VA, visual acuity, VAN, vancomycin.

## Data Availability

Not applicable.
